# Supplementary material for: The assessment of the impact of glistening on visual performance in relation to tear film quality
Source: PLoS One. 2020 Oct 12;15(10):e0240440. doi: 10.1371/journal.pone.0240440 (PMC7549795; doi:10.1371/journal.pone.0240440)

**S1 Fig. The mesopic non-glare (A) and mesopic with glare (B) contrast sensitivity values of two intraocular lenses in different spatial frequencies.** There were no statistically significant differences in any spatial frequencies.


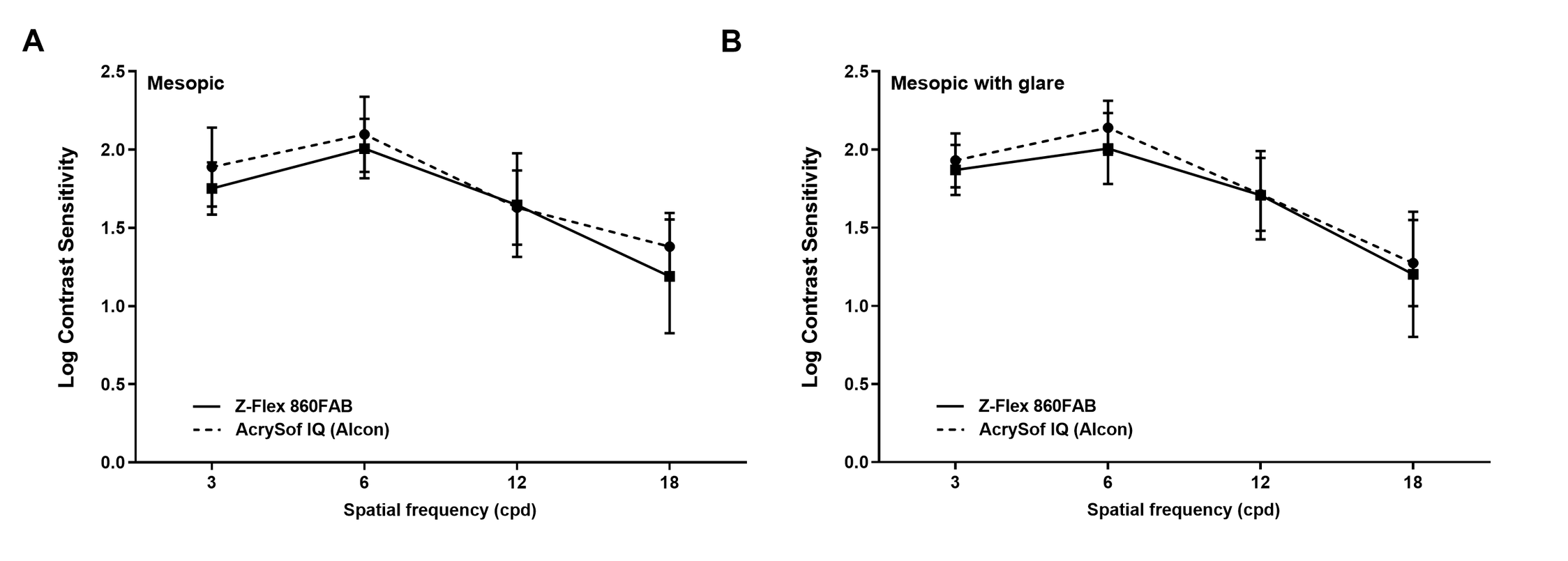

Supplement: S1 Fig — The mesopic non-glare (A) and mesopic with glare (B) contrast sensitivity values of two intraocular lenses in different spatial frequencies. There were no statistically significant differences in any spatial frequencies. (DOCX) [file pone.0240440.s001.docx]
