# Supplementary material for: The assessment of the impact of glistening on visual performance in relation to tear film quality
Source: PLoS One. 2020 Oct 12;15(10):e0240440. doi: 10.1371/journal.pone.0240440 (PMC7549795; doi:10.1371/journal.pone.0240440)
Supplement: S2 Fig — (A) Glistening evaluation with the subjective slit-lamp examination method in eyes implanted with the Z-Flex 860FAB or the AcrySof IQ SN60WF IOL. (B) Objective assessment of glistening by Scheimpflug analysis followed by computer-based image analysis in eyes implanted with Z-Flex 860FAB or the AcrySof IQ SN60WF IOL. (C) Correlation analysis revealed a strong correspondence between the results of the two glistening evaluation techniques. (DOCX) [file pone.0240440.s002.docx]

**S2 Fig. (A) Glistening evaluation with the subjective slit-lamp examination method in eyes implanted with the Z-Flex 860FAB or the AcrySof IQ SN60WF IOL. (B)** **Objective assessment of glistening by Scheimpflug analysis followed by computer-based image analysis in eyes implanted with Z-Flex 860FAB or the AcrySof IQ SN60WF IOL. (C) Correlation analysis revealed a strong correspondence between the results of the two glistening evaluation techniques.**

**
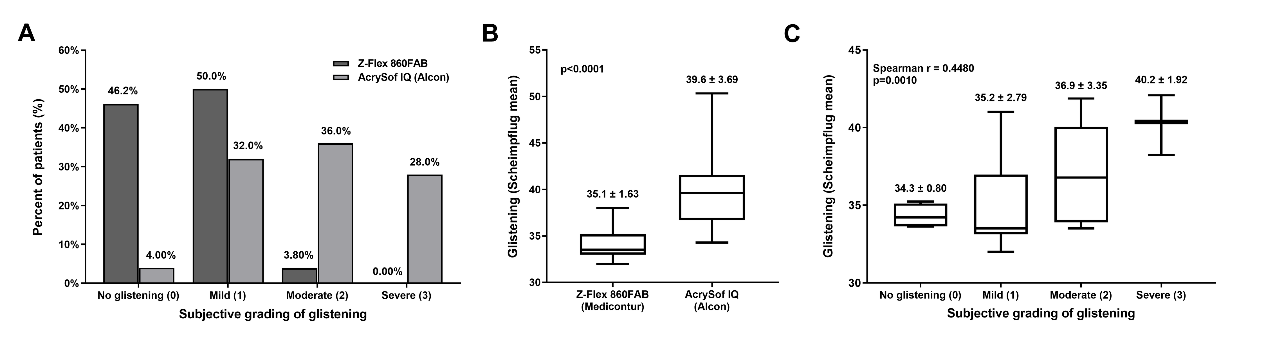
**
