# Supplementary material for: The assessment of the impact of glistening on visual performance in relation to tear film quality
Source: PLoS One. 2020 Oct 12;15(10):e0240440. doi: 10.1371/journal.pone.0240440 (PMC7549795; doi:10.1371/journal.pone.0240440)

**S3 Fig. (A) The quality of the tear film examined by the HD Analyzer and expressed as TF-OSI values of the different IOLs. (B) Correlation between the TF-OSI and BCDVA** (n=23 eyes). TF-OSI=tear film related objective scatter index, BCDVA=best corrected distant visual acuity.


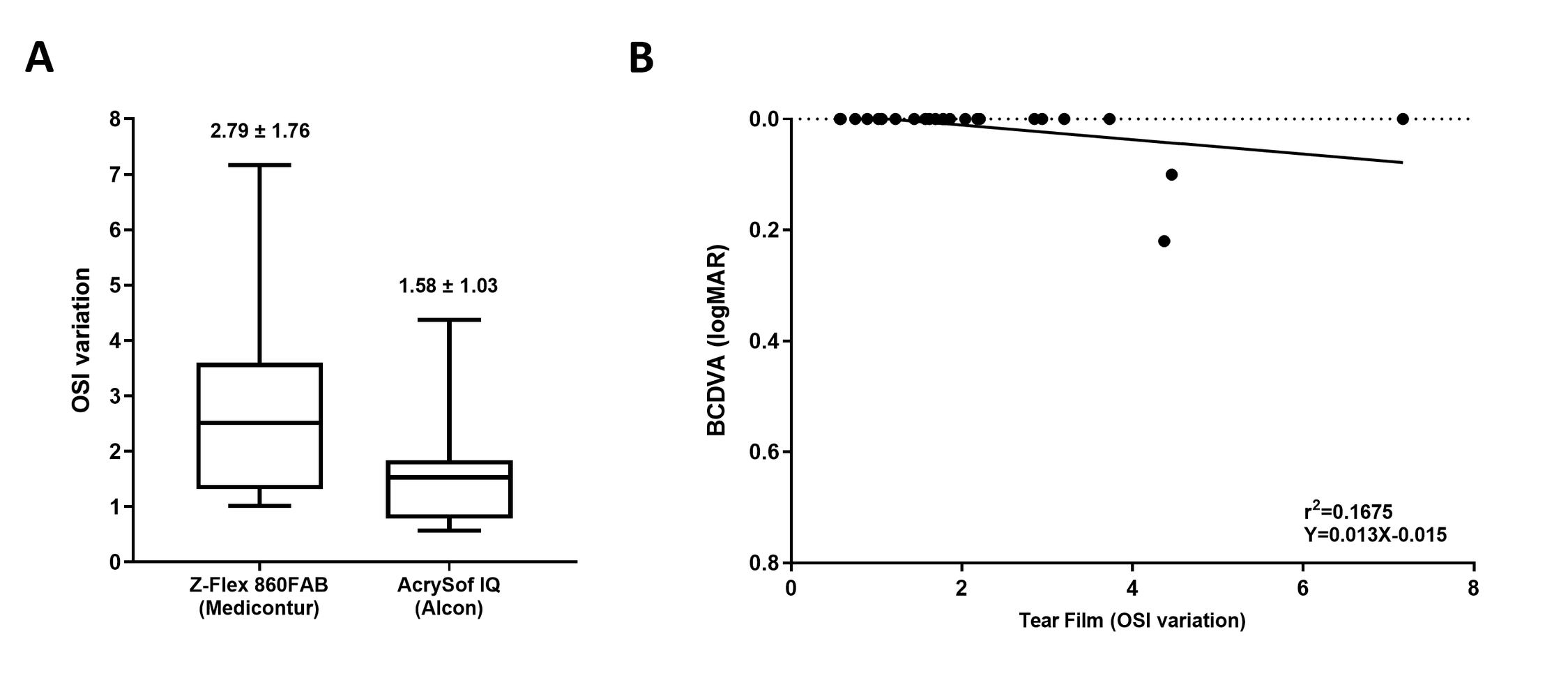

Supplement: S3 Fig — (A) The quality of the tear film examined by the HD Analyzer and expressed as TF-OSI values of the different IOLs. (B) Correlation between the TF-OSI and BCDVA (n = 23 eyes). TF-OSI = tear film related objective scatter index, BCDVA = best corrected distant visual acuity. (DOCX) [file pone.0240440.s003.docx]
