# Supplementary material for: The assessment of the impact of glistening on visual performance in relation to tear film quality
Source: PLoS One. 2020 Oct 12;15(10):e0240440. doi: 10.1371/journal.pone.0240440 (PMC7549795; doi:10.1371/journal.pone.0240440)
Supplement: S1 Table — (DOCX) [file pone.0240440.s004.docx]

**S1 Table. Characteristics of the intraocular lenses used in the study.**

| Characteristic | Z-Flex 860FAB | AcrySof IQ SN60WF |
| --- | --- | --- |
|  |  |  |
| Optic material | Hydrophobic acrylic copolymer  SEMTE | Hydrophobic copolymer acrylate/methacrylate |
| Refractive index | 1.47 | 1.55 |
| Abbe number | 58 | 37 |
| Optic design | Biconvex, 360° square edge^*^,  anterior and posterior aspheric surface | Biconvex, square edges, anterior and posterior aspheric surface |
| Optic diameter (mm) | 6.0 | 6.0 |
| Length (mm) | 13.0 | 13.0 |
| Haptic configuration | Double C-loop | Modified L |
| Haptic angulation (°) | 0°with posterior vaulting | 0° |
| Ultraviolet filter | Yes + blue light filter | Yes + blue light filter |
| A-constant (SRK/T) | 118.9 | 118.7 |
|  |  |  |

^*^ Patented.
