# Supplementary material for: The assessment of the impact of glistening on visual performance in relation to tear film quality
Source: PLoS One. 2020 Oct 12;15(10):e0240440. doi: 10.1371/journal.pone.0240440 (PMC7549795; doi:10.1371/journal.pone.0240440)
Supplement: S2 Table — Demographics, AXL = axial length, UCDVA = uncorrected distance visual acuity, BCDVA = best corrected distance visual acuity, logMAR = logarithm of the minimum angle of resolution, IOL = intraocular lens, D = dioptre. (DOCX) [file pone.0240440.s005.docx]

**S2 Table. Pre-operative datas.** Demographics, AXL=axial length, UCDVA= uncorrected distance visual acuity, BCDVA=best corrected distance visual acuity, logMAR=logarithm of the minimum angle of resolution, IOL=intraocular lens, D=dioptre.

| Demographic | Z-Flex 860FAB | | AcrySof IQ SN60WF | | Significance  (p) |
| --- | --- | --- | --- | --- | --- |
|  | **Mean ± SD** | **Range** | **Mean ± SD** | **Range** |  |
|  |  |  |  |  |  |
| Age (y) | 71.9 ± 5.3 | 64 - 81 | 66.6 ± 8.4 | 50 - 79 | **0.0189** |
| Gender (n) |  |  |  |  |  |
| Female | 17 (77.3%) |  | 12 (60.0%) |  |  |
| Male | 5 (22.7%) |  | 8 (40.0%) |  |  |
| AXL (mm) | 23.65 ± 1.07 | 22.39 - 26.95 | 23.24 ± 0.78 | 21.42 - 24.62 | 0.3381 |
| SPH (D) | 1.03 ± 2.08 | -3.75 - +4.75 | -0.60 ± 2.99 | -8.0 - +2.75 | 0.0736 |
| CYL (D) | 1.18 ± 0.75 | 0.25 - 2.5 | 0.89 ± 1.44 | 0.0 - 5.0 | **0.0285** |
| SEQ (D) | 1.37 ± 1.89 | -2.75 - +5.38 | -0.10 ± 2.75 | -8.0 – +4.75 | **0.0289** |
| UCDVA (logMAR) | 0.68 ± 0.37 | 1.7 – 0.1 | 0.78 ± 0.45 | 1.7 – 0.3 | 0.5456 |
| BCDVA (logMAR) | 0.35 ± 0.36 | 1.7 – 0.0 | 0.48 ± 0.45 | 1.7 – 0.0 | 0.2179 |
| IOL Power (D) | +20.4 ± 2.69 | +12.0 - +25.0 | +21.7 ± 1.98 | +17.0 - +25.0 | **0.0340** |
|  |  |  |  |  |  |
