# Supplementary material for: The assessment of the impact of glistening on visual performance in relation to tear film quality
Source: PLoS One. 2020 Oct 12;15(10):e0240440. doi: 10.1371/journal.pone.0240440 (PMC7549795; doi:10.1371/journal.pone.0240440)
Supplement: S3 Table — m = manifest, D = dioptre, SE = spherical equivalent, UCDVA = uncorrected distance visual acuity, BCDVA = best corrected distance visual acuity, logMAR = logarithm of the minimum angle of resolution. (DOCX) [file pone.0240440.s006.docx]

**S3 Table. Post-operative visual outcomes.** m=manifest, D=dioptre, SE=spherical equivalent, UCDVA=uncorrected distance visual acuity, BCDVA=best corrected distance visual acuity, logMAR=logarithm of the minimum angle of resolution.

| Demographic | Z-Flex 860FAB | | AcrySof IQ SN60WF | | Significance  (p) |
| --- | --- | --- | --- | --- | --- |
|  | **Mean ± SD** | **Range** | **Mean ± SD** | **Range** |  |
|  |  |  |  |  |  |
|  |  |  |  |  |  |
| SPH (D) | +0.51 ± 0.50 | -0.25 - +1.5 | +0.49 ± 0.70 | -0.75 - +2.5 | 0.9988 |
| CYL (D) | -0.36 ± 1.12 | -2.0 - +1.5 | -0.63 ± 0.96 | -3.5 - +0.75 | 0.4058 |
| SEQ (D) | +0.36 ± 0.65 | -1.0 - +1.75 | +0.18 ± 0.70 | -1.5 – +1.5 | 0.3559 |
| UCDVA (logMAR) 4 years postop. | 0.25 ± 0.20 | 0.7 – 0.0 | 0.13 ± 0.15 | 0.5 – 0.0 | **0.0329** |
| BCDVA (logMAR) 4 years postop. | 0.08 ± 0.14 | 0.6 – 0.0 | 0.02 ± 0.06 | 0.2 – 0.0 | 0.0611 |
| UCDVA (logMAR) 6 years postop. | 0.19 ± 0.16 | 0.5 – 0.0 | 0.14 ± 0.17 | 0.6 – 0.0 | 0.3607 |
| BCDVA (logMAR) 6 years postop. | 0.01 ± 0.03 | 0.1 – 0.0 | 0.02 ± 0.06 | 0.2 – 0.0 | >0.9999 |
| Tear-film (OS variation) | 2.79 ± 1.76 | 1.02 – 7.17 | 1.58 ± 1.03 | 0.57 – 4.37 | **0.0449** |
|  |  |  |  |  |  |
